# Dealing With Stress in Cats: What Is New About the Olfactory Strategy?

**DOI:** 10.3389/fvets.2022.928943

**Published:** 2022-07-15

**Authors:** Lingna Zhang, Zhaowei Bian, Qingshen Liu, Baichuan Deng

**Affiliations:** Laboratory of Companion Animal Science, Department of Animal Science, South China Agricultural University, Guangzhou, China

**Keywords:** cat stress, welfare, olfaction, pheromone, chemical signals

## Abstract

Domestic cats are descended from solitary wild species and rely heavily on the olfaction system and chemical signals for daily activities. Cats kept as companion animals may experience stress due to a lack of predictability in their physical or social environment. The olfactory system is intimately connected to the brain regions controlling stress response, thus providing unique opportunities for olfactory strategies to modify stress and related behavioral problems in cats. However, the olfactory intervention of stress in cats has been mainly focused on several analog chemical signals and studies often provide inconsistent and non-replicable results. Supportive evidence in the literature for the potentially effective olfactory stimuli (e.g., cheek and mammary gland secretions, and plant attractants) in treating stress in cats was reviewed. Limitations with some of the work and critical considerations from studies with natural or negative results were discussed as well. Current findings sometimes constitute weak evidence of a reproducible effect of cat odor therapy for stress. The welfare application of an olfactory stimulus in stress alleviation requires a better understanding of its biological function in cats and the mechanisms at play, which may be achieved in future studies through methodological improvement (e.g., experiment pre-registration and appropriate control setting) and in-depth investigation with modern techniques that integrate multisource data. Contributions from individual and environmental differences should be considered for the stress response of a single cat and its sensitivity to olfactory manipulation. Olfactory strategies customized for specific contexts and individual cats can be more effective in improving the welfare of cats in various stressful conditions.

## Introduction

Domestic cats are one of the most popular pets worldwide. At present, people in cities live a fast-paced lifestyle but are in need of companionship. Cats offer an outlet for nurturing with relatively lower maintenance requirements (e.g., some degree of independence, less space, and social commitment) (1). However, cats are not traditionally kept for companionship, and changes in lifestyle and environmental predictability have exposed, especially indoor cats, to many restraints and aversive stimuli. Stress is an important issue in cats with serious health and behavioral consequences (1, 2). Either being part of the normal reaction to aversive stimuli but considered inappropriate by owners or indeed problematic, behavioral problems are among the top risk factors for cats to be relinquished and euthanized in the shelter (1, 3–6). In China, escaped or abandoned cats could contribute to the population of free-ranging cats that exert a huge threat to the local wildlife populations and diversity (7). The study of stress and related behavioral problems in cats has the significance of promoting cat wellbeing, human–cat relationship, and healthier ecosystem as a result of reduced abandonment of owned cats. Management of stress in cats often includes the provision of environmental enrichment, such as hiding enrichment (8–11). Dietary supplementation of functional ingredients and prescribed antidepressants were also reported in pet dogs and cats (12, 13) which are beyond the scope of the current review. Similar to many other carnivore species, cats rely heavily on their olfactory system to explore the physical and social environment. Even now considered a facultatively social species, cats often chose to live a solitary life with enough space and resources (14, 15). Chemical communication is, therefore, involved in many inter-cat activities, such as territory marking, reproduction, and individual recognition (16, 17). The olfactory system can serve as a potential target for the modulation of stress response due to its intimate connection with the central limbic system (18). Olfactory stimuli developed with scientific guidance may provide many opportunities for stress management in cats. By integrating current research on olfactory intervention on different stress markers, the goal of the current review is to evaluate the effectiveness of different olfactory stimuli on the regulation of stress in cats and provide insights into future research directions in this field.

## Stress Response, Triggers, and Behavioral Signs of Stress in Cats

### Triggers of Stress

Cats are constantly adapting to their living environment, detecting and interpreting the various stimuli as either being neutral, positive, or aversive. Aversive stimuli or stressors can be classified into two major categories (i.e., physical and psychological) that are perceived and processed differently in the brain and involve the recruitment of distinctive amygdala and noradrenergic cell groups (19, 20). Psychological stressors, defined as stimuli that exert a threat or are anticipated as threats are indicated as more potent than physical triggers such as body infection or hemorrhage (19). Most of the stressful stimuli in captive cats are situations where either predictability is lacking or the need of a cat is not satisfied (2, 21–23). Some common triggers of stress in cats are summarized in Table 1. For example, the exposure to a novel environment and social interaction (21, 42) and change in caretaking routine (32, 33, 44) is controlled by owners or working staff but not the cat. Cats that have no outdoor access or are in lengthy sheltering may not be meeting their needs of expressing natural behavior and social interaction (4, 58).

**Table 1:** Common behavioral signs and triggers of stress in cats.

**Acute or prolonged stress**	**Behavioral signs**		**Triggers**
Acute stress	Anxious posture, shaking, fast ventilation, fully dilated pupil and flattened ears, tail close to the body, plaintive vocalization (24–27), struggle, motor activity and aggression (27–29), hiding attempt (8–11) Reduced activity level and diversity, including play, exploration, and maintenance behavior such as feeding, drinking, and elimination (25, 26, 30, 31), reduced social affiliation and facial marking (22, 32), occurrence of feigned sleep (33, 34), increased vigilance (35),		Bath (36, 37) Hospital visit (38–40), handling and restraint practices (28, 29) Confinement (27, 41) Novel environment (e.g., entering shelter) (21, 42, 43) New socialization, such as group housing (21, 42)
Prolonged/chronic stress	Sickness behavior, (e.g., vomiting)		Changed caretaking routine (24, 32, 33, 44)
	Anorexia		Long-term sheltering (31)
	House soiling problem (45, 46)	In appropriate elimination (1, 47)	Social conflict, blocked access to the litterbox (1), changes related to litter (47, 48) Chronic disease, such as feline idiopathic cystitis (49)
		Fecal marking (1)	Outdoor and indoor social conflicts (1)
		Urine marking (1, 50)	Lower urinary tract disorders (51), substantial changes in the social and physical environment (1, 49, 50, 52)
	Depression-like symptom (e.g., inactivity)		Long-term sheltering (53)
	Aggression (2, 54–56)		Social conflicts (2, 54, 57), high housing density (43), co-residence with dogs (58) and other cats (59), long-term sheltering (53)
	Stereotypic behavior (e.g., over grooming or self-mutilation, tail biting, and obsessive vocalization) (60, 61)		Stress from chronic disease, environmental and social conflict (1, 60, 61) Frustration from limited outdoor access (58, 61, 62)

### Stress Response

**Figure 1:**
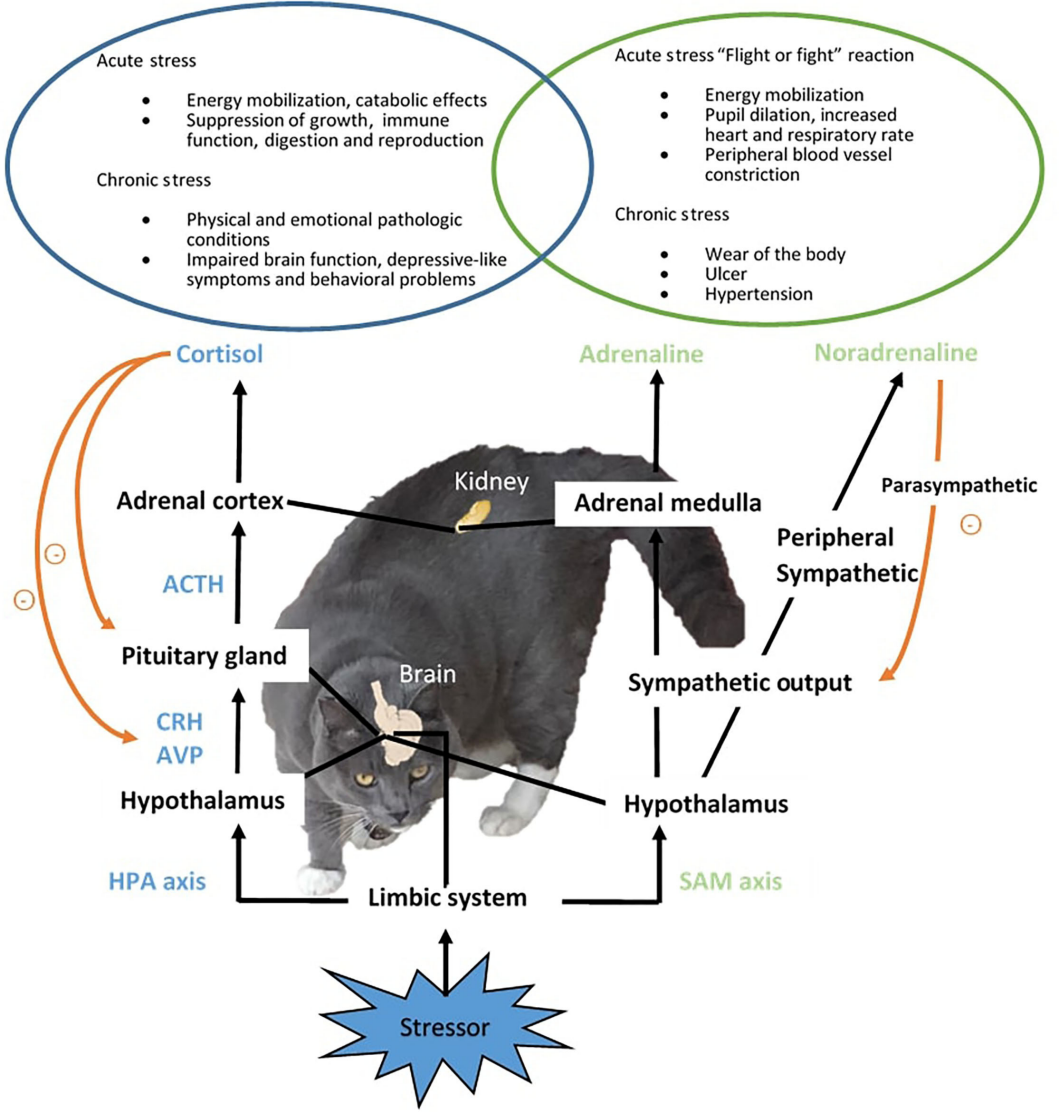
Stress response in cats. Upon the detection of an aversive stimulus or stressor, two major components of stress response, the sympathetic-adreno-medullar (SAM) and hypothalamic-pituitary-adrenal (HPA) axis will be activated, resulting in the release of mediating hormones, including mainly fast acting catecholamines from the SAM axis, and corticotropin-releasing hormone (CRH), arginine vasopressin (AVP), adrenocorticotropic hormone (ACTH) and cortisol from the HPA axis. The stress response coordinates physiological and behavioral changes to assist the restoring of organism homeostasis from the interference of stressors. When adaption is not achieved, sustained stress response can cause physiological and psychological pathological conditions.

A real or perceived stressor in the environment triggers the rapid activation of two major components involved in stress response, the sympathetic-adrenal-medullar (SAM) and hypothalamic–pituitary–adrenal (HPA) axis, and the release of mediating hormones to co-ordinate the physiological and behavioral adaptations and restoring of homeostasis (Figure 1) (2). In the SAM axis, the activation of the posterior hypothalamus stimulates the adrenal gland medulla *via* the splanchnic nerve and causes the release of the fast-acting catecholamines, adrenaline and noradrenaline (NA), which activate the “flight or fight” reaction (36) and mediate the first signs of the stress response such as elevated blood pressure (38) and increased heart rate and respiratory rate (39). The sympathetic output will then be decreased by the parasympathetic nervous system. In parallel, the HPA axis is also activated. Corticotropin-releasing hormone (CRH) is secreted together with arginine vasopressin from the paraventricular nucleus (PVN) in the hypothalamus and acts on the posterior pituitary gland, causing the release of adrenocorticotropic hormone (ACTH) which stimulates the adrenal cortex to release glucocorticoid hormones, predominantly cortisol to the circulation in cats. An increase in cortisol secretion is commonly reported in cats exposed to acute stressors, such as bath (36, 37) and hospital visit (26). The elevated cortisol then exerts negative feedback on the pituitary and hypothalamus to prevent further release of CRH and ACTH. Glucocorticoids affect a vast range of processes pertaining to metabolism, immune function, and brain activity, temporally shutting down systems not emergent for immediate survival, such as digestion and reproduction (2). The response to acute stress declines after successful adaptation and/or the removal of the stressor. In chronic stress where the animal is subjected to prolonged stress response, dysregulation of the HPA axis (33) and pathologies (e.g., ulcer and infection) can occur, resulting in compromised welfare (2, 63). It had been suggested that the common emotions companying the stress response include fear and chronic state of anxiety (2). Animals can also exhibit behavioral disorders due to chronic stress. In humans and rodent species, chronic stress is linked to the development of mental disorders such as depression (18). Monoaminergic neurons such as dopamine (DA), NA, and serotonin (5-HT) that project to the pre-frontal cortex are initially enhanced to keep the function of the pre-frontal cortex low during the “flight or fight” response. Sustained or intermittent stress exhausts the monoamine neurotransmitters and causes a decline in neuron function, which has a series of consequences, in the limbic DAergic neurons causing the loss of pleasure, and in the amygdala and hippocampus resulting in memory and emotional dysfunction. Collectively, the incidence of mental and behavioral disorders is increased when the brain function deteriorates under chronic stress. In aging cats, stress contributes to the development or worsening of cognitive dysfunction (64).

### Behavioral Signs of Stress in Cats

Physiological and behavioral measures, as well as health indicators, are the most important parameters used for evaluating stress in cats (65). However, a comprehensive and valid welfare tool is not currently available. Methodological restrictions and difficulties (e.g., short of non-invasive, field-verified monitoring devices, or testing methods) in obtaining accurate physiological data in various settings have rendered behavior a particularly common welfare-assessing tool (65, 66). Behavioral exhibition of stress in cats is summarized in Table 1. Overall, a preference for concealed areas (i.e., hiding), reduced activity level and diversity, anxious body postures, and aggression are indicative of acute stress in cats (8, 24, 26). Noteworthy, coping style (e.g., reactive or proactive) was reported to impact behavioral responses of individual cats to stressful events or environments such as acute cage confinement (41). Animals with a proactive coping style often exhibit active behavioral responses (e.g., territorial control and aggression) and are characterized by high activation of the SAM axis and low HPA axis activation, while those with a reactive coping style exhibit withdrawal response (e.g., immobility, hiding attempts, and low level of aggression) and had higher parasympathetic reactivity and HPA-axis activation (67, 68), which points out the importance of addressing individual differences when using behaviors to evaluate stress in animals. Behavioral problems developed from prolonged or chronic stress may be less apparent but with increased severity. Depression-like symptoms can occur, such as loss of pleasure seeking, social inhibition, increased aggressiveness, and altered fearfulness, and can negatively impact several sensory perceptions, such as olfaction deficits, blunted taste, and hyperalgesia in humans and other animal species (69). Behaviors indicating learned helplessness (e.g., increased resting/sleeping) have been shown in dogs in lengthy captivity (70). In shelter cats, increased residence time is positively correlated with cats being increasingly inactive and more involved in conflict situations (53). Chronically stressed cats may express behaviors that are either abnormal (e.g., stereotypic behavior) or normal but with altered frequency and/or unwanted (e.g., urine marking) by owners (2, 22). In many cases, several behavioral problems (e.g., elimination disorder, urine marking, and aggression) were exhibited concurrently in one cat (4, 62). The time frame required for animals to be considered under chronic stress is an unanswered question, one agreed upon definition being that the stress has to occur intermittently and persist for weeks or months (66). There is less information on how individual differences impact the cat's adaption and resistance to future stress and the possibility of developing specific behavioral problems with different types of chronic stress. Future studies addressing these issues can be significant for identifying stress-sensitive cats and the early prevention of developing chronic stress in these cats.

## Cat Olfactory System

Similar to many other mammals, cats have at least two olfactory systems to perceive and process the various chemicals existing in their environment (16). Small volatiles that reach the main olfactory epithelium are often inhaled during breathing and detected by the main olfactory system. These chemicals bind to the receptors on the ciliated dendrites of the first-order olfactory sensory neurons in the olfactory epithelium, the axons of which converge onto glomeruli at the main olfactory bulb (MOB). From here, information is conveyed to the primary olfactory cortex *via* the lateral olfactory tract and further spread to other brain regions (e.g., orbitofrontal cortex) *via* the thalamus. The MOB is also connected to parts of the limbic system to control some hypothalamic activities (18). The perception of chemical signals by the accessory olfactory system occurs through the vomeronasal organ (VNO), a pair of liquid-filled sacs located at the roof of the mouth and encased within bony capsules in the septum. It is connected to both nasal and oral cavities *via* the nasopalatine canal. The VNO is suggested to be mainly involved in the detection of water-soluble molecules. Upon investigating the area, cats often sniff and lick the fluid material and exhibit the Flehmen response which opens the nasopalatine canal and allows the passage of fluid-borne molecules to the VNO (16, 71). The chemosensory neurons lie in the epithelium of VNO and send axons to the accessory olfactory bulb (AOB) located in the dorsal-posterior region of MOB. Information from AOB is not sent directly to the cortex but rather to the emotion-controlling limbic system, where neurons mainly connect to the hypothalamic nuclei to control mostly instinctive behaviors and responses (69, 72). It was previously believed that different chemicals were detected exclusively by the VNO or the main olfactory epithelium, but recent research supports that inputs from both olfactory systems are required for the appropriate processing of some social and predator-related chemosensory stimuli (73, 74). Two systems send inputs to separate but adjacent nuclei in amygdala, and the divergent signals are integrated for relay to the basal forebrain regions to initiate behavioral responses (75–77). Information about how chemical signals detected by the olfactory system are processed in the brain is mainly based on rodent and human studies and is quite limited in cats. Nevertheless, the olfactory system and scent communication play critical roles in many cat activities, such as marking and social interactions (17, 71, 78).

## Reducing Stress in Cats: the Olfactory Strategies

Considering its unique intimacy with the limbic system, the olfactory system may serve as a potential target for the intervention of stress response. This is still a matter of debate that warrants further investigation. Olfactory stimuli that induce stress (i.e., from predators and stressed conspecifics) should be avoided (69), while those with comforting or enriching effects may be applied in aversive contexts to reduce stress and improve welfare in cats. The potentially effective stress-reducing and/or enriching scent stimuli for cats are summarized in Table 2.

**Table 2:** Scent stimuli with potential stress-reducing and/or enriching effect for cats.

**Scent name/commercial**	**Source**	**Component**	**Potential function**	**Application evidence**
F3/Feliway™	Cat cheek/sebaceous gland	Oleic acid, azelaic acid, pimelic acid, palmitic acid	Object marking	Putative effect of reducing urine spraying/marking (79), debated efficacy of calming cats at vet clinic and shelter (80, 81)
Appeasing pheromone/Feliway^®^ Multicat or Feliway^®^ Felifriend	Queen mammary sulcus/ skin sebaceous gland during nursing	Oleic acid, palmitic acid, linoleic acid, myristic acid, lauric acid, and stearic acid	Appeasing the queen and kittens	Reduce inter-cat aggression in multi-cat household (82), and also improve cat interaction with co-resident dogs a 6-week testing period (83)
Pedal/ Feliway^®^ Feliscratch™	Cat interdigital area/skin sebaceous gland	Valeric acid, lactic acid and, linoleic acid	Territory marking	Induce scratching when applied to scratching posts (84), more likely due to catnip (85)
Prey odor	Rat, rabbit	Odor mixture	Induce predatory or play behavior	Inconclusive results in captive cats (86, 87)
	Rabbit maternal-neonatal pheromone	2-methyl-2-butenal (2M2B)	Unknown	Improve use of litter box and reduced aggression in pair-housed cats when applied to litter box (88)
Familiar interspecific scent	Mostly from owner	Odor mixture	Comforting effect	Not effective during the strange situation test (89), remains to be tested in other settings
Cat attractant	Plant such as catnip and silver vine	e.g., Neptalactone in catnip; isoiridomyrmecin and dihydronepetalactone in silver vine	Chemical defense against mosquitoes	Inducing play behavior in a proportion of adult cats (90–92), calming cats at clinic together with F3 analog (93), cat habituation is common (86)

### Cat Scent, Signature Mixtures, and Pheromone

Mammalian chemical signals can be classified into pheromones or signature mixtures based on their functions (73). Cats have a number of mechanisms to produce chemical signals, including scent glands throughout the body, and the salivary, fecal, and urinary sources (71). Meanwhile, several marking methods (e.g., rubbing, scratching, and urine marking) that involve the deposit of chemical signals have been described in cats (94). Pheromone is a species-wide chemical signal and belongs to semiochemicals which include also interspecific communicative chemicals (i.e., allomone, kairomone, and synomone). Pheromone is defined as the chemical signal emitted by one individual and elicits a stereotyped behavior or response in the receiving conspecific individual (16, 73). Pheromone communication benefits both emitter and receiver and varies slightly between individuals in the species. Examples of pheromone include the sexual odors given off by queens in estrus (71). Signature mixtures are indicative of an animal's chemical profile, allowing the differentiation between individuals and colonies (73). The anal gland secretions fall into the category of signature mixtures (78). The classification of some chemical signals is debated. For example, it is suggested that the facial cheek secretions should be considered signature mixtures instead of pheromones because the component and content of facial gland secretions vary in cats (95). A better understanding of the functional properties of different chemical signals of cats can provide opportunities for scent strategies in different settings that can reduce stress and improve cat welfare. A series of synthetic chemical products have been developed for context-dependent use in cats.

Rubbing objects and individuals in the environment is an affiliative behavior in cats that allows the deposit of gland secretions for physical and social marking, organizing the environment, and exchange of scents between individuals (14, 71). So far, several chemical signals have been identified from sebaceous secretions of cat facial area, namely facial pheromone F1–5. The major active components of the cheek secretions are volatile fatty acids, such as oleic acid and palmitic acid. It was suggested that cats use F3 for object marking and organization of their environment, and use F4 for allomarking of other individuals (71). Feliway™, a commercial product mimicking F3, was developed for environmental application to reduce stress in cats, the logic being that the artificial pheromone increases the familiarity of the environmental objects and individuals for the cat. Several studies reported an effect of Feliway™ on reducing stress-related urine spraying in cats, as reviewed by Mills et al. (79). However, only one study followed the randomized controlled design and was double-blinded (96). The F3 analog product was also shown to calm cats at the vet clinic although it did not reduce struggling during handling (30, 97), however, its lack of efficacy in a study including a similar scenario was also reported (98). Another study showed that in a shelter environment, salivary cortisol levels did decrease for the majority of cats (75%; 21/28 cats) following 35 days of F3 analog treatment and male cats responded better to the intervention than female cats (99). However, no control group was included in this study. Stress is related to the contraction and recurrence of certain diseases in cats, including the upper respiratory infections as a result of feline herpesvirus (100, 101) and feline idiopathic cystitis (102). Respiratory tract symptoms caused by feline herpesvirus in shelter kittens were reduced in the pheromone-treated group (100), while another study found no effect of Feliway™ on stress scores or incidence of upper respiratory tract infection in adult shelter cats (101). The synthetic facial pheromone was also shown to improve symptoms and stress behaviors in cats with feline idiopathic cystitis (102). Some researchers suggest that there is insufficient evidence for feline facial pheromone product in calming cats (80, 81), given the lack of positive results and limitations with the experimental design in the aforementioned studies. Collectively, supportive evidence exists for the efficacy of synthetic feline facial pheromone in reducing anxiety and stress-related behaviors, such as urine marking. However, randomized and well-controlled studies with more rigorous methodology are encouraged in the future for validating the use of Feliway™ in additional settings.

Secretions from skin glands of the mammary sulcus by queens during nursing were proposed to have a calming effect and appease both kittens and queens, therefore, called appeasing pheromone (71). The commercial version of appeasing pheromone, Feliway™ Felifriends or Multicat, was shown to reduce inter-cat aggression in multi-cat households (82) and also improve cat interaction with co-resident dogs over a 6-week testing period (83). With only two studies currently published, much remains to be learned about the efficacy of Feliway™ Felifriends in promoting amiable social behaviors, e.g., the settings for application and individual differences in their responses to the pheromone treatment. It has been suggested that kittens' early experience with the pheromone (hand-raised orphan vs. queen-raised) might influence their response to the appeasing pheromone (103).

Cats under chronic stress show reduced behavioral diversity; therefore, promoting the expression of natural behavior (e.g., scratching) has welfare potential in stimulating behavioral diversity and reducing anxiety. Cats have sweat glands in the planter pads and interdigital skin of the pedal area. Secretions from these glands were suggested to be involved in scent marking and producing alarming messages (71). Scratching is often exhibited on object surface (e.g., furniture in an indoor environment), particularly on vertical surface by male cats (85, 94), and leaves behind physical and chemical marks in the environment (71, 104). Cats tend to return to the same spot for scratching, suggesting that the visual and scent mark served as a reference point (105). The feline interdigital semiochemical (FIS) product, FeliScratch™, contains FIS and catnip extract. Application of FeliScratch™ on the scratching devices successfully directed scratching from furniture to the provided device in 74% (22/29) of cats (106). In another study with a crossover design, cats scratched more of the scratching post that is treated with FeliScratch™ than the placebo post (84). However, our recent study showed that the efficacy of inducing starching, if any by FeliScratch™, is more likely due to the ingredient of catnip than FIS (85).

Other sources of chemical signals (e.g., urine, feces, and anal gland secretions) in cats have been identified and their potential functions have been investigated (78, 107, 108). Fecal scents, anal gland secretions, and even facial pheromones are technically all signature mixtures for individual identification (78, 95, 107). It is still debated about which source of chemical signals represents the scent of an individual cat. Social buffering that is the presence of a social companion can moderate HPA responses to stress, and the nature of the relationship between individuals will determine whether or not social buffering of stress response will occur (109). Synthetic analogs that often include mixtures of several representing chemicals in set concentrations are probably perceived by the receiving cat as scents of another cat. The question arises as how cats interpret the scent of another cat, as enriching or threatening? Data included in a recent review paper reported that the scent of conspecifics provided as enrichment did not result in much change in shelter cats (110). Therefore, without a better knowledge of the exact effects (e.g., valence) of these chemicals on cats, their welfare application will remain to be validated.

### Other Biologically Relevant Scents

#### Prey and Food Odor

Predatory behavior/hunting is one of the most important natural behaviors in cats. Segments of predatory behavior are also incorporated in play, such as stalking, pouncing, and kicking prey or toys. Play is probably a means of hunting practice in cats, as play with different-sized toys matched the interaction pattern with the prey of different sizes (i.e., mice vs. rats) during hunting, and hunger increased the play intensity and interest in larger toys (111, 112). Several studies have investigated the efficacy of prey odor as environmental enrichment in cats, often including other olfactory stimuli for comparison such as the scent of catnip and lavender (86, 87, 110). Shelter cats exposed to cloth impregnated with catnip or rabbit scent, in general, become less active with more time sleeping and reduced exploring of the environment (86). Promoting inactivity may not always be bad as it is important to differentiate between activities that indicate restlessness and anxiety (e.g., stereotypic pacing) and that are indicative of good welfare (e.g., play). In another study, a wooden cube covered with cloth of rat scent induced sniffing and rubbing of the cube in shelter cats (87). The differences in the sources of prey odor (rat vs. rabbit) and measures (instant behavioral responses to the odor vs. general activity) included may contribute to the disagreement between the two studies. Generally, other than catnip, most scents in these studies including prey odor did not induce much interest and predatory behavior in cats. Olfaction plays a minor role during hunting in cats (113). Providing only odor without the sound or visual stimuli of the prey may not be enough to induce predation-related behaviors in cats. Another study added the rabbit maternal-neonatal pheromone, 2-methyl-2-butenal (2M2B), to a cat litter box and found that the use of the litter box was improved and aggression in pair-housed cats was reduced (88). The mechanism underlying this action of rabbit maternal pheromone on cats requires further investigation.

In addition to the pleasure of feeding and the anticipation of food, the stress-reducing effect of cat food could be achieved through functional diets with anti-oxidative or anxiolytic properties (12, 13), and being an element of enriching tools when served together with food puzzles (114). These benefits are more likely to be mediated by the digestive system and crosstalk between the gut and brain rather than by olfactory pathways. Stress inhibits feeding and can cause food neophobia in cats (22, 33). At present, cats are often fed flavored commercial diets. Increasing the attractiveness of food with palatants may promote feeding and recovery from stressful events (115). Most of the food preferences were evaluated in trained or household cats under normal conditions (116). Food odors or the actual preferred food and palatability enhancers are rarely tested for capacities in improving cat feeding in stressful conditions. Nutrient-enriched water with or without poultry flavor was shown to effectively increase cat water intake over a 44-day testing period when compared to control, with the poultry-flavored water that contains more protein and fat being more potent (117). Future research may investigate the stress relief effect of preferred food ingredients and palatants, and odors on cats.

#### Scent of Familiar Human

Cats kept indoors for companionship can form a close relationship with owners or primary caretakers. Recent studies have shown that cats attach to their owners (118) in a way similar to the relationship between children and parents (119), and that between dogs and owners (120). In the strange situation test, cats on overage exhibited less stress-related behaviors in the strange environment when the owner was present compared to being alone, indicating social buffering of stress response in the presence of the owner; the presence of only objects with the scent of owners (e.g., owner's cloth) was not comforting to the cats (89). This is different from the situation in humans and dogs. The odor of the attached figure reduced stress responses in humans during weak electric shocks (121). An fMRI study reported that the caudate nucleus, a brain region related to rewards and positive experiences in un-sedated dogs, was activated after sniffing the scent of a familiar human, but not of a familiar dog (122). Results from the studies with dogs and cats might not be comparable because of the species differences and also differences in the measured variables and experimental settings. Dogs in the study were well-trained to co-operate and remain still in the fMRI machine; thus, the effect of the scent of a familiar human on dogs was evaluated in a neutral to a positive environment due to extensive training with treats. In the case of the cat study, subjects were exposed to a novel environment, which often induces a strong stress response in cats. The scent from a familiar human may not be as effective as the actual presence of the human. However, this does not exclude the potential application of familiar human scents in other non-testing contexts (e.g., hospital visit and stay in a pet hotel). Future studies may also investigate the effects of scents from other familiar cats or pets on cats from multi-pet households.

### Cat Herbal Attractants and Other Plant-Extracted Oil

Cats are naturally attracted by plants such as catnip (*Nepeta Cataria*) and silver vine (*Actinidia polygama*) and react in a euphoric way. Upon sniffing these plants, cats often exhibit the so-called “catnip response,” which is comprised of species-specific playing behaviors, such as rubbing, rolling on the ground, and kicking the plant source. The iridoid compounds in the plants (e.g., nepetalactone in catnip; isoiridomyrmecin and dihydronepetalactone in silver vine) are the major active ingredients inducing the “catnip response” (91). Depending on studies, about 20–60% of the cat population was reported to respond to catnip and up to 90% respond to silver vine (85, 90, 92). The response is mediated by the main olfactory system instead of the accessory olfactory system (123), and is independent of sex or the presence of gonads in cats, rather the response increases as the cat matures (90, 92, 124). The existence or absence of specific olfactory receptor(s) for the plant chemicals may explain the diverse responses in cats (125). It is not until recently that researchers started to investigate the biological function and the underlying mechanism of cat responses to these plant attractants. Uenoyama et al. (125) reported that nepetalactol, the chemically synthesized major active component in the silver vine, increased plasma β-endorphin levels in cats, potentially through the activation of the central rewarding system as inhibition of μ-opioid receptors blocked the classic rubbing response. This study provided supportive evidence that plants like catnip and silver vine elicit pleasure in cats, and the lack of response in kittens is due to their immature opioid system (126). Cats are believed to be not addicted to these attractive plants (127), because the μ-opioid system is not directly stimulated by exogenous opiates, but by elevated endogenous β-endorphin after the activation of olfactory neurons in response to plant odorants (125). These cat-attractive plants, either served alone or together with other stimuli (e.g., toys or scratchers), have been widely used in cats to enrich their environment and increase behavioral diversity. When applied on scratchers, the attractants can increase the use of the scratchers by cats, thus amplifying the enriching effects of these scratching devices (84, 85).

Still, a few things need to be considered when seeking the use of plant attractants for relieving stress in cats. Studies have shown that responses to catnip in captive black-footed cats and shelter cats waned over the 5-day testing period (86, 128), indicating habituation after continuous exposure. Rotation of different plants and limiting free access to the source may help to maintain the attractiveness and effectiveness of these plants. Researchers have proposed that all cats respond to catnip; the active responders exhibit the classic “catnip response,” while the passive individuals show “sphinx-like position” and reduced vocalization and activity after exposure (92). Uenoyama et al. (125) included only positive silver vine responders in the study for the measurement of plasma β-endorphin. It is unknown if cats also respond passively to silver vine. Future study may investigate the secretion of β-endorphin in these passive cats or previously classified non-responders to determine if euphoric effects are also elicited despite the lack of behavioral manifestations. Negative emotions (e.g., fear) may inhibit the cat's response to these plant attractants (69, 90); thus, their application in settings involving acute stress may be limited. These attractants may be more effectively applied to reduce boredom and anxiety in long-term confinement, such as sheltering and daily household. Cats also show preferences and response variations to different plants, and repeated testing at different time points and with different plants may be necessary to induce the active response in individual cats, thus helping to expand the population of cats that can benefit from the intervention.

In humans and rodent species, plenty of studies exist for the positive effect of the scent of coffee beans and essential oils of lavender, cypress, α-pinene, and thyme linalool on stress-related behaviors and expression of stress markers in the brain (18). Such study is quite limited to cats and the only study included lavender as odor enrichment showed almost no effects on cats (86).

## Discussion and Future Directions

Scent plays an important role in many cat activities and can serve as effective enrichment and stress-reducing tools if properly understood and applied (16). The loss of opportunities for cats to receive and emit chemical signals may affect cat welfare. Cats seem to be naturally comforted by certain conspecific scents, such as the F3 cheek secretions (79) and feline maternal pheromone (82, 83). Therefore, olfactory enrichment is important to both prevent and address stress-related behavioral problems. However, close appraisal of the literature on olfactory manipulation for stress alleviation in cats, especially those focused on commercial chemical signals, often reveals limitations with methodology. Some issues, such as deficient experiment design and lack of negative control, may have contributed to the inconsistent results in the literature. Current findings constitute weak evidence that there is a reproducible effect of therapy of cat chemical signals for stress. Future studies may benefit from the practice of pre-registration whereby primary outcomes and measures are declared in advance, specifying statistical methods to be applied, and making data openly available, which increases the credibility of the research.

The pre-requisite for appropriate application is a better understanding of the biological functions of the stimuli. The introduction of odors with stimulating properties may be enriching and can promote mental health, but it had been suggested that these stimulating odors may cause increased agitation and result in the development of active types of problematic behaviors (e.g., stereotypy) (129). Long-term research in more depth on the impact of these stimuli is needed before firm conclusions can be drawn about their welfare applications (129). Researchers may rely on more advanced and less invasive technologies to capture accurate physiological and neuroendocrinal data which can be particularly helpful in stress evaluation. For example, high-quality testing kits can benefit the measure of cortisol and its metabolites in other sources (e.g., urine and feces, hair, and saliva), thus minimizing the effect of the sampling procedure. Wearable electronic devices, such as monitoring collars, can help to collect physiological parameters such as respiratory rate, heart rate, and heart rate variability (130), even though their use in cats requires further validation. A cognitive bias test may be applied to reflect the animal's emotional state which is central to welfare studies (131, 132). Integrated information from multiple sources (e.g., behavioral, physiological, and neurofunctional) may provide more fruitful results for the assessment of the stress-reducing or enriching effects of a given olfactory stimulus.

In addition to methodological improvements, research should address individual differences, which has long been recognized by scientists working with animals. Cats with different coping styles are a case in point. Instead of being invariant, the stress response is currently considered an array of different physiological and behavioral patterns when all kinds of aversive stimuli are encountered by animals (41, 67, 68). Meanwhile, genetic factors, early experience (e.g., effect of maternal pheromone), and emotional state (e.g., stress and mood) may all contribute to varied susceptibility to olfactory manipulation in animals (69). The variant responses of cats to catnip and other plant attractants bear genetic and mental contributions (90). Therefore, individuality in stress coping style and susceptibility to olfactory manipulation could be particularly relevant in seeking olfactory strategies for stress management in cats.

Research may consider insights into the mechanical basis of how odors work and their perception by the animals. A few hypotheses for the potential odor effect on stress relief had been proposed (69) and warrant future investigation. Odorants could directly act on the stress centers in the brain or have indirect impacts, such as masking effect and associative learning. Odorant concentrations in the air and the amount that reaches the olfactory epithelium can be hard to measure and standardize. High doses of odorants in the air may enter the bloodstream and have pharmacological rather than olfactory effects. Positive/neutral odorants can also compete with aversive odorants for binding sites at the sensory epithelium and have masking effects. Odorants considered pleasant and calming for humans can serve as a distraction from the memory recall of negative experiences. Pleasant elements of odorant may also be tested in animals with the assessment of preference variability. The association of odors with a positive emotional state may explain the stress relief action of appeasing pheromone where the animals associate the odors with the maternal environment and early experience. Studies in pigs also showed that when presented with odors that were fed to the sows during late gestation and lactation, piglets exhibited less stress upon weaning (133). The putative effects of food odor and odors of familiar individuals may also be explained by positive association. The social buffering effect of odors from familiar partners is dependent on the quality of the relationship which reflects the accumulative association with experience of former interactions (109). An alternative hypothesis should be tested as well. For example, it is possible that odors act on the owner instead of having direct effects on the cat. The emotional state of the owner has been shown to impact their interaction style with the cat, the cat's stress and emotion, and its behavioral health (134).

Given the importance of olfaction in regulating cat behavior, olfactory strategies hold a huge potential for treating stress-related problems in cats. However, some of the current findings constitute weak evidence for reproducible effects of odor therapy for stress in cats. A better understanding of the biological functions of the various olfactory stimuli requires a systematic methodological appraisal and the investigation of the mechanisms at play. Future studies should seek improvement in methodology possibly through preregistration of the experiment, take advantage of the advanced measuring techniques, and recognize the importance of addressing cat individuality (i.e., coping style and susceptibility) and the influence of environmental factors.

## Author Contributions

LZ drafted and wrote the manuscript. BD and QL provided conceptual advice and revised the manuscript. ZB worked on substantial modification at all stages of manuscript preparation. All authors contributed to the article and approved the submitted version.

## Funding

This study was supported by the National Key R&D Program of China (Grant No. 2021YFD1300400), the National Natural Science Foundation of China (Grant Nos. 31790411 and 32002186), the Natural Science Foundation of Guangdong Province (Grant No. 2020A1515010322), the Guangdong Basic and Applied Basic Research Foundation (Grant No. 2019B1515210002), the Independent Research and Development Projects of Maoming Laboratory (Grant No. 2021ZZ003), and the Young Lecturer Grant to LZ funded by the Department of Animal Science at South China Agricultural University.

## Conflict of Interest

The authors declare that the research was conducted in the absence of any commercial or financial relationships that could be construed as a potential conflict of interest.

## Publisher's Note

All claims expressed in this article are solely those of the authors and do not necessarily represent those of their affiliated organizations, or those of the publisher, the editors and the reviewers. Any product that may be evaluated in this article, or claim that may be made by its manufacturer, is not guaranteed or endorsed by the publisher.
